# The double-edged sword effect of employee impression management and counterproductive work behavior: From the perspective of self-control resource theory

**DOI:** 10.3389/fpsyg.2023.1053784

**Published:** 2023-02-01

**Authors:** Hui Ni, Yi Li, Yimei Zeng, Jia Duan

**Affiliations:** ^1^Economics and Management School, Wuhan University, Wuhan, China; ^2^Office of Research, Wuhan Business University, Wuhan, China; ^3^Research Institute for Development of Science and Technology, Hubei University of Technology, Wuhan, China; ^4^Dong Fureng Institute of Economic and Social Development, Wuhan University, Wuhan, China

**Keywords:** impression management, self-promotion, ingratiation, counterproductive work behavior, self-control resource depletion

## Abstract

Why do people who seem to be doing well in the workplace occasionally behave badly? Because these employees may be using impression management tactics to create an image. Existing studies have focused on comparing the differences in the use of impression management among different individuals, but cannot explain why a well-behaved individual sometimes behaves badly. Based on the theory of self-control resources, we adopts the survey method of job logs and collects the data of 121 employees. The results show that: (1) the use of self-promotion tactics and ingratiation tactics will promote the depletion of self-control resources. (2) The depletion of self-control resources will encourage employees to engage in counterproductive work behaviors. (3) The effect of self-promotion tactic and ingratiation tactic on the depletion of self-control resources was moderated by emotional intelligence. In the case of high emotional intelligence, this effect is attenuated. And vice versa. (4) Under high emotional intelligence and low emotional intelligence, the indirect effects of self-promotion tactic and ingratiation tactic on employees’ counterproductive work behaviors are significantly different. Our research breaks through the between-individual perspective and illustrates the double-edged sword effect of self-promotion tactic and ingratiation tactic on employee counterproductive work behavior and its mechanism from the internal perspective, which is highly innovative.

## 1. Introduction

Impression management is ubiquitous in organizational life. It is an important aspect of individual career success and has a wide impact on both individuals and organizations (Carlson et al., 2011; Bourdage et al., 2020). Impression management refers to the behavior of employees to create, maintain, protect, or otherwise change the impression of their target objects (Bozeman and Kacmar, 1997; Bolino et al., 2008). For example, in an interview, ingratiating the preferences of the interviewer by promoting one’s past achievements can increase the chances of being hired (Barrick et al., 2009; Joshua et al., 2018). To achieve the goal of impression management, employees usually adopt a variety of impression management tactics. Self-promotion and ingratiation are the two most widely used impression management tactics (Bolino et al., 2008). Self-promotion tactics can help employees draw attention to their good qualities, future plans, or past achievements. Ingratiation tactics can arouse interpersonal attraction and leaders’ preferences (Proost et al., 2010; Gross et al., 2021).

Previous studies have shown that impression management has an important impact on all aspects of organizational work (Barrick et al., 2009). Ingratiation tactics and self-promotion tactics will positively affect employees’ interview performance (Zhao and Liden, 2011) and performance evaluation (Bolino et al., 2006, 2008), teamwork (Turnley and Bolino, 2001), job promotion (Harris et al., 2007), etc. In addition, some studies have also shown that employees who use impression management tactics are less likely to be fired during difficult economic times (Huang et al., 2014). Most studies in the field of impression management focus on the fact that employees who use impression management in their work are more likely to achieve their personal goals (Bolino et al., 2008). These studies enable us to better understand the differences in the use of impression management among different employees and how impression management can help employees achieve success in the organization (Bolino et al., 2016). Unfortunately, these studies cannot explain why impression management might have negative unintended consequences. Although the study from the perspective of between-individual, it can be analyzed that different individuals use impression management tactics to convey positive self-image, so as to achieve individual career goals such as interview and employment, job promotion, and performance evaluation (Bolino et al., 2016). Most studies have been following this research idea, but they ignore the impact of the use of impression management tactic on the internal impact of individuals and the impact on the organization and other members. From the perspective of within-individual, it is seems impossible to explain why some looking good employees in the organization will occasionally engage in counterproductive work behaviors, such as teasing colleagues and deliberately delaying work. To fill this research gap, we believe that the above phenomenon can only be explained by studying the impact of impression management tactic on users’ internal resources from the perspective of within-individual.

In addition, Klotz et al. (2018) believe that even if employees engage in impression management, sometimes it will have adverse effects on colleagues or damage the interests of the organization and other employees. Although impression management has a significant positive impact on employees’ organizational citizenship behavior, people with strong impression management motivation take great care to avoid creating negative images in the eyes of others (Turnley and Bolino, 2001), they will do some organizational citizenship behaviors to *do well* and *look good* (Grant et al., 2009; Grant and Mayer, 2009). It is very likely that such organizational citizenship behavior is just a strategic tool for employees’ impression management, rather than consistent with their inner attitude. What we are curious about is whether employees in impression management engage in behaviors that are beneficial or harmful to the organization.

Furthermore, previous studies have shown that the use of impression management tactics is actually emotional labor. On the one hand, they could be deep acting, the sincere expression of emotions that align with the heart. On the other hand, it can also be surface acting, dealing with and expressing emotions that are inconsistent with the inner mind (Moon and Hur, 2011; Deng et al., 2020). In impression management, employees’ behavior and inner thoughts may be inconsistent. According to the theory of self-control resources, individuals need certain self-control ability when they make behaviors that violate their true attitudes and mental states, while this self-control depends on limited self-control resources. When this resource is consumed to a certain extent, individuals will fall into a state of ego depletion. In the state of ego depletion, employees’ self-presentation ability and self-management will become ineffective (Vohs et al., 2011; Yam et al., 2014). When employees’ self-control resources are depleted, they are more likely to produce unethical and counterproductive work behaviors (Christian and Ellis, 2011; Gino et al., 2011), such as making fun of colleagues, being late for work, etc. In addition, the use of impression management tactics is also related to personality traits (Bourdage et al., 2012). Turnley and Bolino (2001) studied the effect of self-monitoring on impression management behavior. This study expands the research on the influence of personal characteristics on impression management. According to relevant studies, individuals with high emotional intelligence can better understand the environment and choose effective impression management tactics, while individuals with low emotional intelligence tend to choose ineffective impression management tactics (Raman et al., 2016). Therefore, emotional intelligence also has a contingency impact on the effect and results of impression management tactics.

This study examines the effects of self-promotion and ingratiation impression management tactics on employees’ counterproductive work behaviors from an internal perspective. In the workplace, the decisions of superiors have a significant impact on the careers and benefits of their subordinates. The impression management tactic of employees often takes the superior leader as the target object (Bolino et al., 2016). This study takes manager-targeted self-promotion (MTS) and manager-targeted Ingratiation (MTI) as the research objects. According to the self-control resource theory (Baumeister and Heatherton, 1996), the key role of self-control resource depletion in impression management tactic and employee counterproductive work behavior is proposed. In addition, we integrate the between-individual perspective and introduce emotional intelligence as a moderating variable to study the boundary conditions under which impression management influences employees’ counterproductive work behaviors through self-control resource depletion. Compared with the existing researches, our research perspective is more fine-grained and the research content is also highly innovative.

## 2. Theoretical analysis and hypotheses development

### 2.1. Influence of self-promotion tactic on self-control resource depletion

Manager-targeted self-promotion (MTS) means that an employee promotes its ability, experience and achievements to the superior leader so that can be recognized by the leader. In this way, leaders can trust that the employee is competent for a certain job or position (Bolino et al., 2008). MTS can improve leaders’ recognition of employees’ abilities (Kristof-brown et al., 2002; Karam et al., 2016), will enable employees to obtain higher performance ratings, promotion, and get ahead in the organization (Casciaro and Lobo, 2005; Pfeffer et al., 2006). However, MTS does not always achieve its intended goals. When employees advertise their abilities to their leaders, they over-emphasize the positive results, that is, they over-brag and promote themselves. Such behavior may be perceived by leaders as conceited rather than competent (Turnley and Bolino, 2001). Employees need to make extra efforts and not deviate from the original goal of MTS (Berman et al., 2015). Therefore, the self-control ability of employees is necessary. Self-control refers to the ability to override or change one’s internal reactions and the tendency to interrupt undesirable behaviors (such as impulsivity) (Tangney et al., 2004).

In the theory of self-control resources, individual state self-control depends on limited resources, which will be consumed by previous self-control behaviors (Ridder et al., 2012). According to the model proposed by Baumeister et al. (1998), individual self-control comes from self-control resources, which will be lost over time, just like muscles will become tired after a period of exertion. Thus, after people engage in self-control behaviors, their capacity for further self-control is exhausted, leading to decreased self-control in subsequent behaviors. When using self-promotion tactics, employees need to control the frequency and degree of using MTS in order not to exaggerate and make themselves appear conceited. This process inevitably consumes internal self-control resources of employees. Accordingly, hypothesis 1a is proposed as follows:

*H1a*: Self-promotion tactics targeting superiors have a positive effect on self-control resource depletion.

### 2.2. Influence of ingratiation tactic on self-control resources depletion

Manager-targeted ingratiation (MTI) refers to the fact that an employee flatters the superior leader and does what the leader likes to do so that the employee is liked by the leader (Bolino et al., 2008). Ingratiation can be considered as a series of interpersonal communication influence tactics, which can enhance interpersonal attraction and eventually gain the affection and goodwill of others (Huang et al., 2014). Flattery are widely used in MTI, including agreeing with and approving of the leader’s views, expressing consistency with the leader, so as to gain the leader’s preference. Social psychology research shows that individuals usually prefer people who are similar to themselves, which is called *me-like effect*. However, MTI is not always able to achieve its goal (Swider et al., 2011). When employees use MTI inappropriately, they are more likely to be identified as a brown-noser than a likable employee. Therefore, when using MTI, employees are required to have certain self-control ability. Secondly, in MTI, agreeing with the leader, flattering and other behaviors require employees’ self-control. Because these behaviors are not necessarily the truest thoughts and feelings of employees (Yam et al., 2016). For example, when a superior tells a joke in a public place, employees should act funny even if they do not feel funny inside. In particular, these surface behaviors consume self-control resources (Schmeichel and Vohs, 2009). In the process of using ingratiation tactics, employees are required to show sincerity (Leary and Allen, 2011). Employees must make an effort to appear genuine to avoid being perceived by their bosses as having ulterior motives. This is because leaders are usually suspicious of praise and flattery from junior employees (Bolino and Turnley, 1999). Therefore, in order to make the behavior of flattery and praise in MTI appear sincere and from the heart, it will inevitably consume the internal self-control resources of employees. Therefore, hypothesis 1b of this study is as follows:

*H1b*: Ingratiation tactics targeting superiors have a positive effect on self-control resource depletion.

### 2.3. Influence of self-control resource depletion on employees’ counterproductive work behavior

Counterproductive work behavior (CWB) refers to the behaviors that employees voluntarily violate organizational norms and purposefully harm the interests of organization members (Robinson and Bennett, 1995; Berry et al., 2007). This kind of behavior includes counterproductive work behaviors against colleagues and organizations, which can be summarized as follows: Property damage (theft, injury, abuse), production damage (absence, tardiness, unnecessarily long breaks, deliberate slowing down of work, time theft), personal damage (harassing colleagues, making fun of colleagues), and political damage (favoritism, gossip, passing the blame; Yam et al., 2017). It takes a certain level of self-control resources to avoid employee counterproductive work behaviors (Bazzy and Woehr, 2017). Self-control ability can help employees suppress desires, emotions and impulses (Bazzy and Woehr, 2017). After the depletion of self-control resources, the self-control ability of employees will be weakened. Existing studies show that individuals lacking self-control resources are difficult to resist the temporary benefits brought by unethical behaviors (Hagger et al., 2010), thus making employees fall into counterproductive work behaviors. For example, Counterproductive work behaviors such as lateness and absence can make employees get a short rest. Self-control resources can be restored through rest and relaxation (Tyler and Burns, 2008). When self-control resources are restored to a higher level, employees can effectively control and manage their inner impulses and unethical thoughts. In the absence of self-control resources, employees will find it difficult to avoid these behaviors (Ren et al., 2014). Therefore, high self-control resources can reduce counterproductive work behaviors (Yan et al., 2014).

Employees who use MTS and MTI consume their self-control resources by suppressing the differences between their real thoughts and actual actions. For example, employees who adopt the ingratiation tactic still need to overcome their true feelings and agree with their leaders even if they do not sincerely agree. The ability of self-control is weakened, and thus the ability of self-control in subsequent behaviors is weakened (Hagger et al., 2010). In terms of behavior, they are more dishonest, less helpful to others, and become more aggressive and impulsive (Mead et al., 2009). Self-control is one of the most important factors influencing employees’ counterproductive work behaviors. Employees with a high degree of self-control will have less counterproductive work behaviors (Denson et al., 2011). Therefore, we propose

*H2a*: The self-promotion tactic targeting the superior has a positive impact on employees' counterproductive work behavior, and self-control resource depletion plays a mediating role in this process.

*H2b*: The ingratiation tactic targe1920ting the superior has a positive impact on employees' counterproductive work behaviors, and self-control resources play a mediating role in this process.

### 2.4. The moderating effect of emotional intelligence

In the study of impression management, scholars believe that the style or ability of users of impression management tactics should be considered (Higgins et al., 2003). The concept of Emotional Intelligence (EI) originated from the social intelligence proposed by Thorndike’s (1920). Emotional intelligence is *the ability to monitor your own and others’ feelings and emotions, discern them, and use that information to guide your thoughts and actions*. According to the conceptual connotation of emotional intelligence, it can be decomposed into four dimensions: (1) *The mood perception*, that is, to identify the ability of self and others’ emotions. (2) *The use emotion to promote thinking*, namely the ability to use emotional. (3) *Understanding emotions*, namely the ability to understand the relationship between emotions. (4) *Emotion management*, namely the ability of adjust own and others’ emotions (Mayer et al., 2004).

Individuals with high emotional intelligence can more accurately understand organizational situations (Lopes et al., 2004) and thus choose more effective impression management tactics. In other words, individuals with high emotional intelligence are better at applying the impression management tactics of MTS and MTI. Moreover, individuals with high emotional intelligence are better at using the emotions of others and themselves to guide their own behavior. Hence, employees with high emotional intelligence can perceive the emotions of leaders more effectively when using MTS and MTI tactics, thus to adjust the frequency and degree of using MTS and MTI tactics. Employees with high emotional intelligence do not need to be constantly in a state of self-promotion and ingratiation, thus reducing the depletion of self-control resources. Therefore, employees with high emotional intelligence will experience less depletion of self-control resources in the use of impression management tactics (Moon and Hur, 2011). EI reflects how individuals are able to make adaptive responses to their surroundings. Employees with high emotional intelligence are better able to adapt to the work environment (Andrei et al., 2016). These employees can get more spiritual and material support from their colleagues and leaders, thus increasing their resources. The individuals with a high level EI would be less likely to experience emotional exhaustion/burnout than would individuals with a low level of EI.

Individuals with high emotional intelligence incorporate emotions into their thoughts and identify the emotions of themselves and others. Employees with high emotional intelligence use emotional labor to manage the emotions of themselves and others, and thus have more intensive emotional labor. According to the research, emotional intelligence has a positive relationship with emotional labor (surface behavior). Individuals with higher emotional intelligence will have more surface behavior dimensions of emotional labor (Raman et al., 2016). Therefore, it may be easier to consume employees’ internal resources and make them in a state of resource depletion.

Studies on emotional intelligence have shown that employees with high emotional intelligence are less likely to engage in unethical behaviors, such as counterproductive work behaviors. Employees with high emotional intelligence are better at using positive emotions in daily work, rather than spending them on things that may damage the organization (Yang and Diefendorff, 2009). The accuracy of employees with high emotional intelligence about their feelings enables them to effectively understand and respond to these feelings (Dong et al., 2014). In addition, strong emotional regulation ability will motivate employees with high emotional intelligence to make positive efforts and reduce the negative consequences caused by their lack of self-control resources. Individuals with high emotional intelligence can actively monitor and adjust destructive behaviors triggered by the depletion of self-control resources (for example, making fun of colleagues, stealing company property, etc.) (Dong et al., 2014). Therefore, compared with employees with low emotional intelligence, employees with high emotional intelligence have less counterproductive work behaviors (Clercq et al., 2014). Based on the above discussion, this paper proposes a set of competing hypotheses about the moderating effect of emotional intelligence.

*H3a*: Emotional intelligence has a negative moderating effect on the effect of MTS and MTI on employees' counterproductive work behaviors.

*H3b*: Emotional intelligence has a positive moderating effect on the effect of MTS and MTI on employees' counterproductive work behaviors.

**Figure 1:**
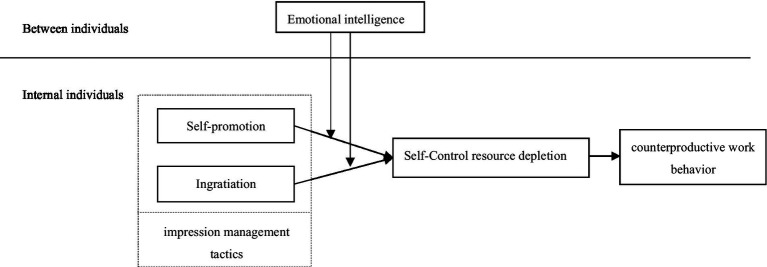
The theoretical framework of the study.

Based on the above theoretical basis and research hypotheses, we draw the theoretical framework of this study as shown in Figure 1.

## 3. Research design

### 3.1. Data collection

According to the theoretical analysis, impression management is essentially a kind of emotional labor. Employees in the service industry often need to conduct impression management to make customers have a good impression on the company. Therefore, we took 155 employees of a large bank in Wuhan as the subjects of the survey, and a total of 140 sets of questionnaires were collected. After excluding 19 sets of questionnaires with serious deficiency, a total of 121 sets of valid paired questionnaires were obtained among individuals and 1,210 valid data were obtained within individuals, and the effective recovery rate of the questionnaire was 78.06%. The questionnaire included two parts: the first part was filled in by the subjects before the first working day to collect the data of individual variables (emotional intelligence). The second part is the work log of 10 working days. In order to avoid homologous variance, the investigator asked the subjects to fill in the questionnaires of MTS, MTI, self-control resource depletion and employee counterproductive work behavior at three different time points every day. The questionnaires at time point 1 (MTS and MTI questionnaires) are filled in by the employees during the morning lunch break every day to describe the work feelings of the subjects during this period of time. The questionnaire (Self-control resource depletion) at time point 2 was filled in by the subjects before they left work in the afternoon and described their work feelings during this period of the afternoon. The questionnaire at time point 3 (Employee counterproductive work behavior) was filled by the subjects after they returned home in the evening, describing the work behavior of the day. These job log questionnaires were used to collect data on individual variables. We will questionnaire envelopes and numbered, 1 each envelope contains notes on completing the questionnaire (Description of the requirements for filling in the questionnaire and Confidentiality agreement that the information will not be disclosed) and 1 copy of the first working day before fill in the questionnaire and 10 content as well as job log (including the questionnaire of three time points).

The reason why we adopts the method of work log to collect data is that: (1) work log can reflect the daily self-control state of employees and the dynamic changes of employees’ behaviors in the workplace. (2) The collected data is consistent with the actual experience of employees, which is helpful to better demonstrate the relationship between MTS and MTI and employees’ counterproductive work behaviors. (3) The survey lasted for 10 working days, that is, 2 weeks, and the survey period was based on the literature on diary research (Johnson et al., 2014). They believed that the fluctuation of work experience of sample participants could be summarized in 2 weeks.

### 3.2. Variable measurement

In this study, independent variables, dependent variables, mediating variables and moderating variables were measured using scales developed by western scholars and widely recognized by scholars. At the same time, the Chinese version of the scale, which has been sampled in China and obtained reliability and validity support, was used whenever possible. For the English version of the measurement tools, we adopt a strict back translation method to ensure the accuracy of translation. The variables in this study are shown in Table 1.

**Table 1:** Variable description and measurement.

Dimension	Variable description		code
Internal individuals (First level)	Control variables	Weekday	*Weekday*
		Weather	*Weather*
	Antecedent variables	Self-promotion tactic	*MTS*
		Ingratiation tactic	*MTI*
	Outcome variable	Counterproductive work behavior	*CWB*
	Mediating variable	Self-control resource depletion	*SCRD*
Between individual (Second level)	Control variable	Gender	*Gen*
		Education background	*Edu*
	Moderating variable	Emotional intelligence	*EI*

#### 3.2.1. Self-promotion tactic aimed at superiors

MTS is measured by the scale developed by Bolino and Turnley (1999), which contains 4 items. For example: *Talk to the leader about your experience or education with pride*; *Let your leader know your talents or qualifications*; *Let your leader know your value in the organization*; *Let your leader know your accomplishments* (Cronbachα coefficient was 0.938).

#### 3.2.2. An ingratiation tactic aimed at superiors

MTI was measured by the scale prepared by Bolino and Turnley (1999), which contained four items. For example: *Compliment your leader so he/she will think you are a likable person*; *Show your friendliness by caring about your leader’s personal life*; *Compliment your leader’s accomplishments so he/she will think you are a nice person*; *Show your friendliness by catering to your leader’s personal preferences* (Cronbachα coefficient was 0.924).

Self-control resource depletion. Five items of Johnson et al. (2014) were used to measure SCRD, such as *I feel my energy is slowly losing*; *My mind is not focused*, *I feel like my willpower is gone* (Cronbachα coefficient was 0.898).

#### 3.2.3. Counterproductive work behaviors

For counterproductive work behaviors, we require employees to self-report using the 19-item scale proposed by Bennett and Robinson (2000), because these behaviors are usually carried out privately by employees without the knowledge of team leaders. In addition, in past studies, employee counterproductive work behaviors were usually self-reported. Seven of the 19 items measure counterproductive work behavior directed at other members of the organization, such as *teasing someone at work*; *be rude to someone at work*; *Falsify invoices to claim more money than you spend at work*; *Taking property at work without permission*. Twelve items measure counterproductive work behaviors directed at the organization, such as *falsifying invoices to claim more money than you spent at work*; *Taking property at work without permission*. This scale has been used in the Chinese context (Fehr et al., 2017). (Cronbachα coefficient was 0.963).

#### 3.2.4. Emotional intelligence

Nineteen items were measured by Dong et al. (2014). For example: *I can read people’s facial expressions to detect their emotions in the present moment*, *When someone I know is in a bad mood, I can quickly calm him down and make him feel better, I can know what people are thinking even if they do not say it directl, I can handle most frustrating problems*. (Cronbachα coefficient was 0.810).

#### 3.2.5. Control variables

This study selected relevant control variables at the two levels of within-individual and between-individual. The weather of the day and the working day are selected as control variables within the individual, and both of them are reflected in the work log. Working days are from Monday to Friday, 10 days in total. There are three kinds of weather: sunny, cloudy and rainy. According to existing studies, weather often affects individual self-control emotions and states, and working days affect employees’ working states. Taking this as a control variable is conducive to the study of the relationship between impression management and self-control resource depletion (Lanaj et al., 2014). Gender and educational background were selected as control variables between the individual.

### 3.3. Data management and analysis

We use SPSS to manage data. In order to make the test results more reference, we use the Mplus software to conduct the mediation effect of bootstrap. Because the main effect of the model is the witin-individual layer variable, the moderating effect is the between-individual level variable. Therefore, we use the hierarchical linear model (HLM) to test the moderationg effect. The first layer is MTS and MTS, self-control resource depletion, employee counterproductive work behavior, and the second is emotional intelligence. Finally, R software was used to conduct moderated mediation tests on the model.

## 4. Empirical results

### 4.1. Descriptive statistics and correlation analysis

Table 2 shows the mean, standard deviation and correlation coefficient of each variable. The correlation coefficient between the variables showed that MTS was positively correlated with CWB (*r* = 0.33, *p* < 0.01), MTI was positively correlated with CWB (*r* = 0.30, *p* < 0.01), and SCRD was positively correlated with CWB (*r* = 0.16, *p* < 0.01). These correlation coefficients provide preliminary support for the subsequent hypothesis of this study (Table 3).

**Table 2:** Descriptive statistics and correlation coefficient matrix of variables.

Variables	Mean	Std. Dev within	Std. Dev between	1	2	3	4	5	6	7	8	9
1. Weekday	3.81	1.92	–	1								
2. Weather	1.65	0.63	–	0.175	1							
3. MTS	2.21	0.93	–	−0.08	−0.02	1						
4. MTI	2.34	1.02	–	−0.07	−0.02	0.66**	1					
5. SCRD	2.91	0.94	–	−0.05	−0.01	0.08**	0.13**	1				
6. CWB	1.70	0.98	–	−0.04	−0.01	0.33**	0.30**	0.16**	1			
7. Gen	1.74	–	0.45	−0.01	−0.03	−0.08*	−0.13*	0.08	0.02	1		
8. Edu	2.53	–	0.49	−0.03	−0.01	−0.19	0.10**	0.03	0.09	0.04	1	
9. EI	3.29	–	0.38	−0.01	−0.04	0.13**	−0.07	−0.17**	−0.02	−0.11	0.01	1

Variables 1–6 are within-individual (*n* = 1,210). Variables 7–9 are between-individual (*n* = 121). **p < 0.01; *p < 0.05.

**Table 3:** Variance component analysis.

Variables	within-individual variance (*e*^2^)	between-individual variance (*r*^2^)	Percentage of variance within individuals
MTS	0.414	0.463**	47.21%
MTI	0.437	0.560**	43.83%
SCRD	0.483	0.410**	54.09%
CWB	0.490	0.471**	50.99%

*e*^2^ represents the within-individual variance and, *r*^2^ the between-individual variance in the variable. Percentage of within-individual variance was computed as the *e*^2^/(*e*^2^ + *r*^2^). *p ≤ 0.05; **p ≤ 0.01; ***p ≤ 0.001.

### 4.2. Variance component analysis

In order to prove that it is meaningful to analyze the influence of MTS and MTI on employees’ counterproductive work behavior from the perspective of between-individuals, we conducted variance component analysis to test the proportion of variance of MTS, MTI, self-control resource depletion and employees’ counterproductive work behavior from within-individual and between-individual. The null model of HLM is used to analyze the first-layer (within-individual) variables one by one, and the results are shown in Table 4. MTS is derived from the internal variance of 47.21%, MTI is derived from the internal variance of 43.83%, self-control resource depletion is derived from the internal variance of 54.09%, and employee counterproductive work behavior is derived from the internal variance of 50.99%. The results in Table 4 also reflect that even for the same individual, the use of MTS and MTI tactics changes with their own state. Therefore, it is feasible and necessary to examine the relevant results of impression management tactic from the within-individual perspective.

**Table 4:** Results of mediation analysis.

	Counterproductive work behavior					Control resource depletion	
	M1	M2	M3	M4	M5	M6	M7
Control variables							
Week	0.164**	0.152**	0.149**	0.109	0.113**	0.537**	0.534**
Weather	−0.040	−0.014	−0.019	−0.010	−0.017	−0.046	−0.044
Independent variable							
*MTS*		0.321**		0.318**		0.052*	
*MTI*			0.290**		0.285**		0.094*
Mediating variables							
*SCRD*				0.082*	0.067*		
*R* ^2^	0.029	0.130	0.112	0.127	0.108	0.296	0.302
*N*	1,210	1,210	1,210	1,210	1,210	1,210	1,210

**p < 0.01; *p < 0.05

### 4.3. The influence of self-promotion tactic and ingratiation tactic on the depletion of self-control resources

Table 4 shows the data analysis results of the bootstrap mediation effect test conducted by Mplus. Hypothesis 1a states that MTS has a positive effect on self-control resource depletion. According to Model 6, MTS have a significant positive effect on self-control resource depletion after controlling for weather and working days (*r* = 0.052, *p* < 0.05), hypothesis 1a was verified. Hypothesis 1b states that MTI has a positive effect on self-control resource depletion. Model 7 showed that MTI had a significant positive effect on self-control resource depletion after controlling for weather and working days (*r* = 0.094, *p* < 0.05), the results supported hypothesis 1b.

### 4.4. Influence of MTS and MTI on employees’ counterproductive work behavior

In order to test the influence of MTS and MTI impression management tactics on employees’ counterproductive work behavior, and test the mediating role of self-control resource depletion in the influencing process, models 1–5 were constructed. The results of model 2 in Table 4 show that MTS has a significant positive impact on employees’ counterproductive work behaviors (*r* = 0.321, *p* < 0.01). Model 3 shows that MTI also has a significant positive effect on employees’ counterproductive work behaviors (*r* = 0.290, *p* < 0.01). Therefore, the significant relationship between MTS and MTI on employee counterproductive work behavior is verified. The significant relationship between MTS and MTI on self-control resource depletion was supported in the tests of hypotheses 1a and 1b. Model 4 showed that MTS still had a positive effect on employee counterproductive work behavior after adding self-control resource depletion (*r* = 0.318, *p* < 0.01). The depletion of self-control resources also had a positive effect on employees’ counterproductive work behaviors (*r* = 0.082, *p* < 0.05). Model 5 showed that MTI had a positive effect on employees’ counterproductive work behavior after adding self-control resource depletion (*r* = 0.285, *p* < 0.01). Self-control resource depletion also had a positive effect on counterproductive productive behavior (*r* = 0.67, *p* < 0.05). It can be seen that, after controlling the mediating variables, although MTS and MTI still have a positive impact on employees’ counterproductive work behavior, the correlation coefficient is slightly reduced, that is, the impact intensity becomes weaker. Results from Models 1 to 5 indicate that hypotheses 2a and 2b are validated.

### 4.5. The moderating effect of emotional intelligence

**Figure 2:**
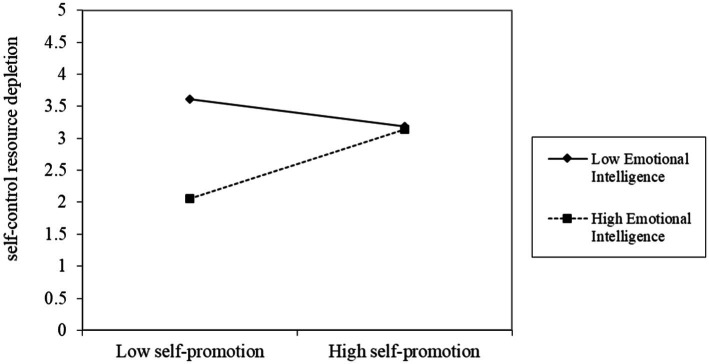
The effect of MTS moderated by emotional intelligence.

**Figure 3:**
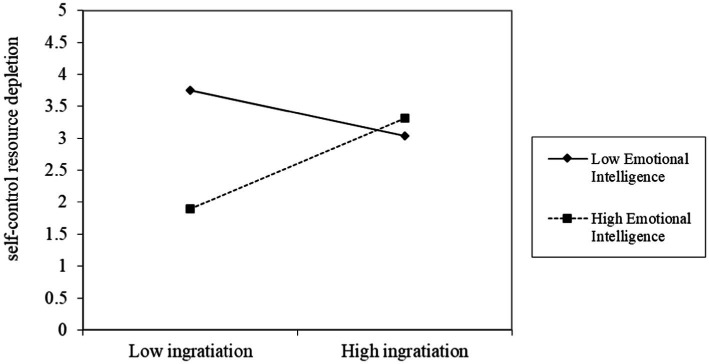
The effect of MTI moderated by emotional intelligence.

In the moderating effect test, we centralized the interaction terms between MTS and MTI and emotional intelligence. We tested the moderating effect of emotional intelligence in the first stage, and constructed Models 8 and 9 with self-control resource depletion as the dependent variable (as shown in Table 5). Model 8 inputs control variables and MTS in the first layer and control variables and emotional intelligence in the second layer. The results showed that the interaction term between MTS and emotional intelligence had a positive effect on self-control resource depletion (*r* = 0.376, *p* < 0.01). It indicates that when employees with high emotional intelligence use MTS tactic, their depletion of self-control resources increases, but the depletion of self-control resources of employees with high emotional intelligence is lower than that of employees with low emotional intelligence. Hypothesis 3a has been preliminarily verified (as shown in Figure 2). Model 9 inputs control variables and MTI in the first layer and control variables and emotional intelligence in the second layer. The results showed that the interaction term between MTI and emotional intelligence had a positive effect on self-control resource depletion (*r* = 0.535, *p* < 0.01), indicating that the self-control resources of employees with high emotional intelligence increase when they apply the modular MTI tactic, but the depletion of self-control resources of employees with high emotional intelligence is lower than that of employees with low emotional intelligence. Hypothesis 3b is initially supported (see Figure 3).

**Table 5:** Results of moderation analysis by HLM.

	Self-control resource depletion	
	M8	M9
Control variables(within-individual)		
Week	−0.037**	−0.031**
Weather	0.033	0.046
Independent variable		
*MTS*	0.161**	
*MTI*		0.178**
Control variables(between-individual)		
Gen	0.123	0.153
Edu	0.051	0.062
Moderating variable		
EI	−0.401**	−0.397**
MTS*EI	0.376**	
MTI*EI		0.535**

**p < 0.01; *p < 0.05.

According to the above analysis results, we plotted the moderating effect of the interaction between MTS and emotional intelligence, and the interaction between MTI and emotional intelligence on self-control resource depletion (see Figures 2, 3). The moderating effect of emotional intelligence can be more intuitively understood through Figures 2, 3.

Figure 2 shows that in the case of high emotional intelligence, the MTS tactic consumes less self-control resources. However, in the case of low emotional intelligence, MTS will consume more self-control resources. In the process of the change from low to high degree of MTS tactic use, the self-control resource depletion of employees with low emotional intelligence decreased. However, it is still higher than the self-control resource depletion of high emotional intelligence.

Relevant studies have shown that individuals with high emotional intelligence are better at using their own and others’ emotions and guiding their own behavior by changes in others and the environment. This process requires more effort and internal resources such as self-control and emotion. Therefore, individuals with high emotional intelligence have an increasing depletion of self-control resources (Raman et al., 2016). However, individuals with low emotional intelligence tend to be more consistent with their behavior and inner thoughts, and the state of impaired self-regulation ability caused by the depletion of self-control resources can be recovered by rest or relaxation (Tyler and Burns, 2008). Therefore, individuals with low emotional intelligence have decreased self-control resource depletion. However, employees with high emotional intelligence still consume less self-control resources than those with low emotional intelligence when using MTS tactic. In the case of high emotional intelligence, the use of MTS tactic leads to less depletion of self-control resources, thus weakening the influence of MTS tactic on employees’ counterproductive work behaviors.

Additionally, Figure 3 shows that under high emotional intelligence, the MTI tactic consumes less self-control resources. Under low self-emotional intelligence, the MTI tactic consumes more self-control resources. Therefore, under of high emotional intelligence, the effect of MTI tactic on employee counterproductive work behavior is weakened through self-control resource depletion.

Although hypothesis 3a and hypothesis 3b were initially verified, in order to further test, we used R software to comprehensively examine the indirect moderating effect of emotional intelligence. One standard deviation above the centralized mean represents high emotional intelligence, and one standard deviation below the centralized mean represents low emotional intelligence. As shown in Table 6, when emotional intelligence is high, MTS tactic has a significant indirect effect on employees’ counterproductive work behaviors through self-control resource depletion (indirect effect = −0.031, 95% CI = [0.002, 0.126]). In low emotional intelligence, the indirect effect of MTI on employees’ counterproductive work behaviors through self-control resource depletion was not significant [indirect effect = −0.002, 95% CI = (−0.072, 0.011)]. However, the difference between high and low emotional intelligence was also significant [difference = 0.029, 95% CI = (0.001, 0.191)]. The test results are consistent with hypothesis 3a. Therefore, hypothesis 3a is fully validated.

**Table 6:** The indirect effect of *MTS.*

MTS (*X*1)	Control resource depletion (*M*)		Employee’s counterproductive work behavior (*Y*)	
	Path a	Path b	Indirect effect	95%CI
	*X*1-*M*	*M*-*Y*	Path a*Path b	
High emotional intelligence (+1 s. d.)	0.305	0.101	0.031	(0.002, 0.126)
Low emotional intelligence (−1 s. d.)	0.016	0.101	0.002	(−0.072, 0.011)
Difference	0.289	0	0.029	(0001, 0.191)

Table 7 shows that when emotional intelligence is high, MTI tactic has a significant indirect effect on employee counterproductive work behavior through self-control resource depletion [indirect effect = 0.037, 95% CI = (0.009, 0.139)]. Under low emotional intelligence, the indirect effect of MTI tactic on employees’ counterproductive work behaviors through self-control resource depletion was significant [indirect effect = −0.003, 95% CI = (−0.081, −0.002)]. The difference was also significant [difference = 0.040, 95%CI = (0.013, 0.214)]. The test results are consistent with hypothesis 3b. Therefore, hypothesis 3b is fully verified.

**Table 7:** The indirect effect of *MTI.*

MTI (*X*2)	Control resource depletion (*M*)		Employee’s counterproductive work behavior (*Y*)	
	Path a	Path b	Indirect effect	95%CI
	*X*2-*M*	*M*-*Y*	Path a*Path b	
High emotional intelligence (+1 s. d.)	0.383	0.095	0.037	(0.009, 0.139)
Low emotional intelligence (−1 s. d.)	0.028	0.095	−0.003	(−0.081, -0.002)
Difference	0.355	0	0.040	(0.013, 0.214)

## 5. Conclusion and discussion

### 5.1. Conclusion

This study not only emphasizes the study of impression management tactic from the perspective of withen-individual, but also integrates the perspective of between-individuals. Our conclusions are as follows: (1) when employees use MTS and MTI tactics for impression management, they will accelerate the depletion of self-control resources. During this period, employees’ self-control ability is weakened. When self-control is reduced, employees are more likely to engage in counterproductive work behaviors. (2) Employees with high emotional intelligence consume less self-control resources when using MTS and MTI tactics. They produce fewer counterproductive work behaviors. (3) Emotional intelligence can moderat the mediating effect of self-control resource depletion. Under high emotional intelligence, the influence of self-control resource depletion on counterproductive work behavior is weakened.

### 5.2. Theoretical significance

This study has the following important theoretical contributions to impression management research.

First, through theoretical analysis and practical investigation, this study proved that MTS and MTI impression management strategies had different mechanisms of consuming employees’ self-control resources. MTS strategy should be used at the right time and occasion to avoid being perceived as arrogant or conceited by leaders. For example, in the Chinese context, leaders are more likely to recognize humble employees. MTI strategy should be used in a sincere manner to prevent leaders from thinking it has ulterior motives and hypocrisy (Peck and Hogue, 2018).

Second, this study breaks through the inter-individual perspective of existing research mainstream, and studies the impact of MTS and MTI impression management strategies on employees’ internal resources from the perspective of individuals. Employees’ self-control ability comes from self-control resources, which will be consumed after a period of use. When employees’ self-control resources are consumed to a certain extent and their self-control ability is damaged, they will be in a state of self-depletion. In the state of ego depletion, employees lack the ability to resist temptation and suppress their inner true feelings, so they are more likely to make counter-productive behaviors to the organization and colleagues. This includes anti-production behaviors against colleagues (such as making fun of colleagues, being rude to colleagues, etc.), and anti-production behaviors against the organization (such as taking company property, intentionally being late, etc.). The use of MTS and MTI strategies will consume employees’ internal self-control resources, which will weaken employees’ self-control ability in the subsequent work.

Third, the study explains why some “nice looking” employees occasionally engage in unethical behavior. This study enriches the relationship between impression management strategies and organizational variables. However, previous studies paid more attention to the benefits brought by the use of impression management strategies for employees’ career, and rarely paid attention to the impact of impression management strategies on employees, their organizations and colleagues. Through theoretical and empirical tests, this study shows that when employees use MTS and MTI strategies, their self-control resources will be lost and their self-control ability will be weakened. It is difficult for employees in the state of self-depletion to resist the temporary benefits brought by the counterproductive production behavior, so as to commit the counterproductive production behavior and damage the interests of other employees and the organization. This explains why some well-behaved employees occasionally behave badly.

Finally, we examines the moderating effect of emotional intelligence. Although we adopts the within-individual perspective, it also draws on the between-individual perspective adopted by existing studies to examine the role of emotional intelligence, an individual characteristic of employees. When employees with high emotional intelligence use MTS and MTI tactics, they consume less self-control resources and thus produce less counterproductive work behaviors. Because employees with high emotional intelligence are good at recognizing, regulating and using the emotions of others and themselves, and have higher social skills. When the behavior is inconsistent with the inner truth, the individual will suffer less internal shock, and therefore consume less self-control resources. However, employees with low emotional intelligence are not good at recognizing, regulating and using their own and others’ emotions, and cannot effectively apply impression management tactics. When using MTS and MTI tactics, more self-control resources need to be consumed to express behaviors that are inconsistent with the heart. Therefore, employees with high emotional intelligence have better effects when using MTS and MTI tactics. When performing surface behaviors, individuals with high emotional intelligence consume less self-control resources, thus reducing employees’ counterproductive work behaviors under impression management. Hence, our research enriches the contextual factors of MTS and MTI tactics on self-control resource depletion (Maher et al., 2018).

### 5.3. Practical enlightenment

For managers, first of all, they should not only be aware of whether employees are carrying out self-promotion and ingratiation impression management tactics, but also identify factors in the organization that promote employees’ impression management behaviors, such as approaching performance appraisal or limited resources. Managers should identify these factors and strive to create a relaxed and open organizational culture that minimizes employees’ impression management motivation. Secondly, managers should guide the employees who make self-promotion and ingratiation behaviors out of the motivation of impression management. It should also be noted that the employee’s self-control resources are being depleted. At this time, managers can let employees do some easy work or rest, so that employees’ self-control resources can be replenished and restored, so as to reduce employees’ counterproductive work behaviors.

For employees, when they engage in MTS and MTI behaviors out of the motivation of impression management, they should be aware that their self-control resources will be consumed, thus weakening their self-control ability. When the self-control ability is weakened, not only the effect of impression management tactic will be greatly reduced, but also some behaviors that are detrimental to the organization and others may be done. When these behaviors detrimental to the organization and others are known by leaders and colleagues, the positive image created by the previous impression management will also be destroyed, and the depletion will ultimately outweigh the gain (Long, 2021). Secondly, employees should have a good understanding of their own emotional intelligence. If emotional intelligence is high, they will not only have a better effect on the use of impression management tactics, but also consume less self-control resources in the process of using impression management tactics. If your own emotional intelligence is low, it is best not to use impression management tactics.

### 5.4. Limitations and future research directions

This study also has some limitations. (1) Variables of this study were all from the self-evaluation reports of employees, and there was no other evaluation data. In reality, however, most employees are reluctant to describe their counterproductive work behaviors truthfully. Although the time point method was adopted in the research design to avoid the influence of homologous variance, future research could try to use the combination of self-rated reports and other-rated reports in data collection. (2) To avoid the effect of homologous variance, the working log of this study is divided into three time points to fill in, lead to collect the MTS and MTI tactic from the employees behave in the morning, and self-control resources depletion measuring work is the change of the psychological state of a day, work hard to rule out the afternoon rest time employees make self-control the interference of resource recovery. (3) In this study, the control variables at within-individual are weather and working days, while the control variables at the between-individual level are gender and educational background, with few control variables. Variables such as age and working years in the unit may also have a certain impact on the correlation coefficient and significance of the variables studied in this paper.

Based on the above potential shortcomings, future research can be expanded from the following aspects. (1) MTS and MTI are two of the five impression management tactics, along with exemplification, supplication, and intimidation. Researchers can study the impact of the other three impression management tactics on employees themselves and the organization from an individual internal perspective (Al-Shatti and Ohana, 2021). (2) We studies self-promotion tactics and ingratiation tactics aimed at superiors, but not all impression management tactics target leaders (Kang et al., 2022). In real life, there are many impression management tactics aimed at colleagues. For example, self-promotion with colleagues as the target, employees publicize their own ability in front of colleagues, and communicate their competence information to leaders through colleagues, is also a widely used impression management tactic. Researchers can study the relationship between impression management tactics that target co-workers and organizational variables. (3) The influence of impression management tactic on employees is not only the depletion of self-control resources, but also the depletion of emotional resources. The mechanism between impression management tactic and employee counterproductive work behavior needs to be further explored. (4) The moderating effect of impression management on employees’ counterproductive work behavior still has a lot of room for exploration. The moderating effect of this study in the first stage, in the second stage whether there are moderating variables and which moderating variables, still need to be further studied.

## Data availability statement

The original contributions presented in the study are included in the article/supplementary material, further inquiries can be directed to the corresponding author.

## Ethics statement

Ethical review and approval was not required for the study on human participants in accordance with the local legislation and institutional requirements. Written informed consent from the participants was not required to participate in this study in accordance with the national legislation and the institutional requirements.

## Author contributions

All authors listed have made a substantial, direct, and intellectual contribution to the work and approved it for publication.

## Conflict of interest

The authors declare that the research was conducted in the absence of any commercial or financial relationships that could be construed as a potential conflict of interest.

## Publisher’s note

All claims expressed in this article are solely those of the authors and do not necessarily represent those of their affiliated organizations, or those of the publisher, the editors and the reviewers. Any product that may be evaluated in this article, or claim that may be made by its manufacturer, is not guaranteed or endorsed by the publisher.
